# Lab-on-a-Disc Platform for Automated Chemical Cell Lysis

**DOI:** 10.3390/s18030687

**Published:** 2018-02-26

**Authors:** Moo-Jung Seo, Jae-Chern Yoo

**Affiliations:** College of Information and Communication Engineering, Sungkyunkwan University, Suwon 440-746, Korea; mtothej92@skku.edu

**Keywords:** chemical cell lysis, Lab-on-a-Disc, laser module, pressure resistant valve, phase change material sheet

## Abstract

Chemical cell lysis is an interesting topic in the research to Lab-on-a-Disc (LOD) platforms on account of its perfect compatibility with the centrifugal spin column format. However, standard procedures followed in chemical cell lysis require sophisticated non-contact temperature control as well as the use of pressure resistant valves. These requirements pose a significant challenge thereby making the automation of chemical cell lysis on an LOD extremely difficult to achieve. In this study, an LOD capable of performing fully automated chemical cell lysis is proposed, where a combination of chemical and thermal methods has been used. It comprises a sample inlet, phase change material sheet (PCMS)-based temperature sensor, heating chamber, and pressure resistant valves. The PCMS melts and solidifies at a certain temperature and thus is capable of indicating whether the heating chamber has reached a specific temperature. Compared to conventional cell lysis systems, the proposed system offers advantages of reduced manual labor and a compact structure that can be readily integrated onto an LOD. Experiments using Salmonella typhimurium strains were conducted to confirm the performance of the proposed cell lysis system. The experimental results demonstrate that the proposed system has great potential in realizing chemical cell lysis on an LOD whilst achieving higher throughput in terms of purity and yield of DNA thereby providing a good alternative to conventional cell lysis systems.

## 1. Introduction

Over the last decade, development of integrated and inexpensive point-of-care (POC) devices for rapid diagnostics has been on the rise owing to miniaturization and automation of analytical protocols based on microfluidics. The obvious advantages of the use of microfluidic devices include reduced sample consumption, enhanced efficiency, and fast reaction times [1,2]. The success of microfluidics in POC applications largely depends upon integration of the principal operation onto a monolithic device. However, the transition from simple independent microfluidic devices to a complete microfluidic POC system capable of performing the fundamental processes of bio-assay protocol is challenging.

Specific to POC applications, improvements to present systems are required to ensure proper integration of microfluidic functions and reduction of fabrication costs; this is in addition to the necessary adaptation of bio-assays to a microfluidic format. In bio-assays, finding and analyzing information regarding genetic and/or disease characteristics is essential [3,4]. Cell lysis, also called cell disruption, is a process that breaks cell membranes open thereby facilitating access to intracellular substances, such as DNA, proteins, and other components for further analysis [5,6]. As the first procedure of cell pretreatment, cell lysis plays a crucial role in obtaining intracellular components, the quality of which directly influences subsequent DNA extraction and amplification [3,7].

Recently, a variety of microfluidic devices have been proposed and demonstrated to perform cell lysis. Cell lysis techniques, based on mechanical, biological, and chemical methods, applied to microfluidic devices have been widely investigated [8,9,10,11,12]. Mechanical methods of cell lysing, such as sonication or freeze-thaw cycles, are the most common ways to lyse cells based on their nature, but these methods are most likely to break DNA [13]. Biological methods often employ lysozymes or bacteriophages. Depending upon the microorganism used, biological methods are labor intensive and may cause damage to nucleic acids and proteins [14]. Chemical methods make use of buffers containing detergents to increase the solubility of lipids and proteins thereby creating pores within the membrane [3]. Commonly used lysis buffers include proteinase K, sodium dodecyl sulfate (SDS), and Triton X-100. Although the chemical method is a relatively slow technique, it is very simple and economical, only marginally damaging to DNA, and highly suited for use in proof-of-concept studies [10].

Amongst numerous microfluidic technologies available, this study proposes the design of a centrifugal microfluidic chip, also known as Lab-on-a-Disc (LOD), which is regarded as one of the most outstanding platforms in microfluidics [15,16,17,18,19]. Typical centrifugal micro-devices perform a set of microfluidic operations, such as liquid transport, metering, aliquoting, mixing, and valving through rotational-speed control [20,21,22,23,24]. Accordingly, such devices have the advantage of being able to control the fluid through use of a single motor to generate the force required for fluid propulsion thereby eliminating the need for an external pump and multiple laboratory instruments. Since fluid control is exclusively regulated by the centrifugal force, the overall process becomes simpler and faster. Implementation of the analytical protocol is based on the use of both capillary and vinyl valves [25,26,27]. Through use of the proposed design, the authors expect to prevent leakage and exercise control over liquid flow with regard to centrifugal microfluidics.

Although many integrated LOD platforms have already been demonstrated for use in biochemistry and whole-blood immunoassay processing [28,29,30,31,32], their heating portion of the assembly has failed to achieve any automation owing to leakage issues. Particularly, in the heating portion of the assembly, previously reported systems have failed to achieve any automation owing to leakage issues. In the proposed system, all microfluidic functions required for execution of the complete assay protocol have been integrated in a simple and quick-to-fabricate polymer-based LOD device manufactured by a carving machine. Additionally, a valve made of black ethylene vinyl acetate (EVA) was used in order to facilitate stable heating.

Through complete integration of a molecular assay for Salmonella species Identification, the objective of this study is to demonstrate the implementation of robust microfluidic functions. Accordingly, the materials, fabrication means, and assembly strategies have been selected to meet requirements for further realistic applications of the device. Phase change material sheet (PCMS) and an illuminance sensor have been used in the proposed system, performing fully automated cell lysis with a combination of chemical and thermal methods. Use these materials enable ease of fabrication, automation of the heating process, and accurate sensing of temperature changes. This proposed platform is expected to be equally applicable to other LOD automation platforms that require wireless heating parts to be fully integrated.

## 2. Materials and Methods

### 2.1. Processing System

**Figure 1:**
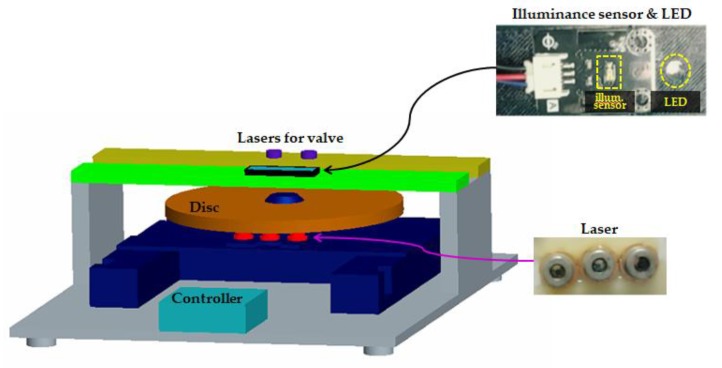
System for Lab-on-a-Disc and testing.

The proposed design of the LOD system is depicted in Figure 1. The design is essentially based on the concept of a CD-ROM used in a computer. The system is equipped with two laser modules. One is for valve control and the other is for conducting cell lysis. The former module serves to block the inlet and vent channels of lysis chamber by two lasers (QL80T4HD-Y, QSI, Cheonan, Korea). Meanwhile, the latter module, made of three lasers positioned to fit the chamber size, serves as a heating source for cell lysis. In order to automate this heating process during cell lysis, a laser controller for heating and maintaining temperature is placed in the lower part of the system. An illuminance sensor (TEMT6000, Vishay, Selb, Germany) is located directly above the laser module (Figure 1). All valves are also operated by laser control. The system laser modules for valve operation and the lysis heating process and the illuminance sensor are centered on the disc. An LED is attached to the side of the illuminance sensor to illuminate the upper PCM layer of the disc. The PC-based laser pulse width modulation (PWM) control is achieved by detecting the varying intensity of the heating process.

### 2.2. Disc Fabrication

The thermally controlled disc platform, capable of performing three parallel sample processes, was fabricated by laminating together five layers comprising two double-sided adhesive films (Tesa 4928, Tesa, Norderstedt, Germany; thickness: 0.125 mm) and three polycarbonate layers. The top and bottom polycarbonate layers were each 1.2-mm thick, and the middle polycarbonate layer measured 4 mm in thickness. The CAD model of the disc was designed using ProEngineer software. Layers of polycarbonate were carved using a computer numeric control (CNC) machine. The top layer combines the PCM structure with the black EVA valve and consists of vent holes for the lysate chamber. The lysis and lysate chambers are located in the middle layer. The lysis chamber (volume = 840 µL) was sufficiently large to handle typical sample solution volumes of 470 µL. The lysate chamber (volume = 750 µL) was positioned away from the center of the disc on account of the sequential progression of the liquid under the action of the centrifugal force.

### 2.3. Preparation of Bacterial Strain and Reagents

Salmonella typhimurium (Tanzania, original ID 73-37671, IB 5379) was used as the target strain to demonstrate the cell lysis operation and liquid control performance of the proposed system. The salmonella sample was inoculated in 25-mL tryptic soy broth and incubated overnight. Subsequently, the sample was subcultured once with the same medium grown to an OD600 value of 1.0, and made aliquoted into cultured salmonella samples measuring 1 mL each in volume. Simultaneously, PBS was washed twice with harvesting and reducing supernatants of the aliquots. To obtain the lysis sample, the amount of supernatant used in the third harvesting was reduced to 250 µL. 200 µL of binding buffer and 20 µL of proteinase K were then added to comprise a total sample volume of 470 µL. Here, the enzyme proteinase K, which is usually activated at 60 °C, will free the DNA from its histones around which DNA is tightly wrapped during the cell lysis. The lysis operation performed on the disc at 60 °C lasted for 10 min, and the lysate was delivered to the lysate chamber. 400 µL of the lysate was pipetted out and passed into a spin column tube that is used to purify DNA from cell debris and chaotropic agents present in the lysis reagent. The purifying procedure was performed following the manual enclosed in the kit (Accuprep^®^ Genomic DNA extraction kit, K-3032, Bioneer, Korea). The solution in the column tube was first washed at 13,000 rpm for 1 min. The column tube was then changed, 500 μL of washing buffer 1 was added, and the solution was centrifuged the same way as in the earlier step. The above step was then repeated using washing buffer 2, and the solution was centrifuged once more to reduce any reagents on the wet membrane. 200 μL of the elution buffer was subsequently added to the solution in the column tube, which was then incubated for 5 min at room temperature. Finally, the column tube was changed, and the solution was centrifuged for 1 min at 13,000 rpm to complete the purification stage. The purified solution was then analyzed by checking the ratio of optical density at DNA and protein absorbance wavelengths of 260 and 280 nm, respectively.

### 2.4. Experimental Procedure of the Disc for DNA Lysis

Table 1 presents the step-by-step sequence of DNA lysis performed using the proposed LOD system. The mechanical system was comprised of a motor (QSI, Cheonan, Korea) to generate a required rpm and, thus, the centrifugal force. To avoid liquid leakage of even the smallest amount, the LOD was made to stably rotate for 30 s, as specified in step 2 in the table. This is done to move all remaining DNA in the channel to the lysis chamber, a major part of which is accomplished in step 1. Laser modules for EVA valve and cell lysis were in operation for each of these processes. In particular, the former was also used in the step 6, which comprises opening of the vinyl valve. The lysate liquid was transferred to the lysate chamber in all amounts owing to the high rpm (5000 rpm) of the system. The entire sequence of processes is completed in about 14 min, including 10 min of the main lysis process.

### 2.5. Ethylene Vinyl Acetate Used as a Valve

This section describes the operation of the DNA lysis heating process and design of the component where the process is accomplished. The chamber, where the sample liquid enters and is heated, vent channel, and inlet channel were fabricated to facilitate completion of the DNA lysis heating process on the LOD device itself. For liquids, the kinetic energy of the particles forming the liquid is increased by the heat being added, and the particle motion becomes active, which causes the liquid volume to increase. The volume change of liquid increases the pressure in the chamber, which implies that the liquid in the chamber now has enough force to flow out of the heating chamber. This explains the tendency of the sample liquid to flow out of the disc when heated. Leakage of even the smallest amount of liquid should be avoided because it may contain certain amount of DNA, which, in turn, may lead to degradation of the DNA yield during extraction and amplification. To address these problems arising due to sample leakage, black hot-melted adhesives based on EVA are used to design the valves.

**Figure 2:**
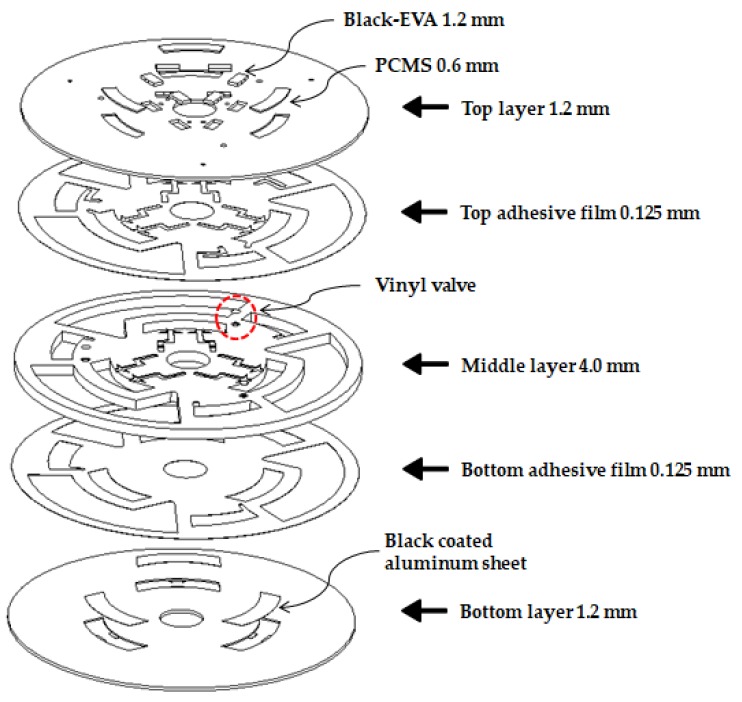
Three layer assembly drawing of Lab-on-disc (LOD). The three layers are made of polycarbonate (PC) plates and bonded with a double-sided adhesive.

**Figure 3:**
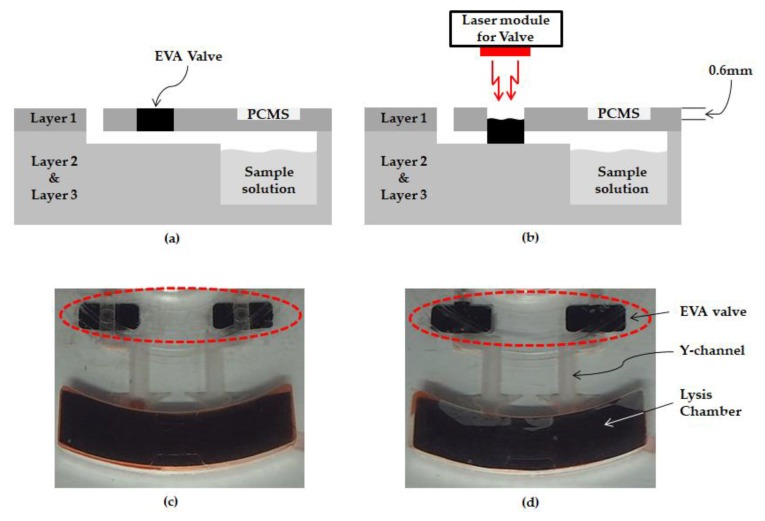
Ethylene vinyl acetate (EVA) valve operation (**a**) side view when EVA valve is open (**b**) side view when EVA valve is closed (**c**) bottom view when EVA valve is open (**d**) bottom view when EVA valve is closed.

Two EVA valves are designed to be incorporated into top layer with a thickness of 1.2 mm, each located in the inlet and vent channel, as seen in Figure 2 and Figure 3a. The release sheet is first placed on the heating plate followed by placement of the layer-1 disc on this sheet. The requisite amount of EVA is then applied at a designated location. Subsequently, as the temperature of the heating plate is lowered, EVA tends to solidify on account of its high melting point of roughly 80 °C. Subsequent machining of the assembly is performed in accordance with disc adhesion, and formation EVA valve is, thus, completed. The energy from the laser incident on the EVA is converted to heat thereby causing EVA to melt, as shown in Figure 3b, and block the channel passage. As a result, the liquid cannot leak out to the chamber unless it is heated to the critical temperature. In order to figure out the critical temperature that the EVA valve can endure, an experiment was performed, wherein the chamber temperature was increased until the liquid began to flow out through the channel. In the proposed design of the disc, only the lysis chamber was sequentially heated by the heating plate. By making use of the heating plate, the durability of the EVA valve was verified. While increasing the temperature of the heating plate, the temperature was assumed to indicate EVA valve durability, which was considered to have ceased upon detection of any liquid flowing through the inlet and/or vent channels.

### 2.6. PCM Used as Illuminance Intensity Change

**Figure 4:**
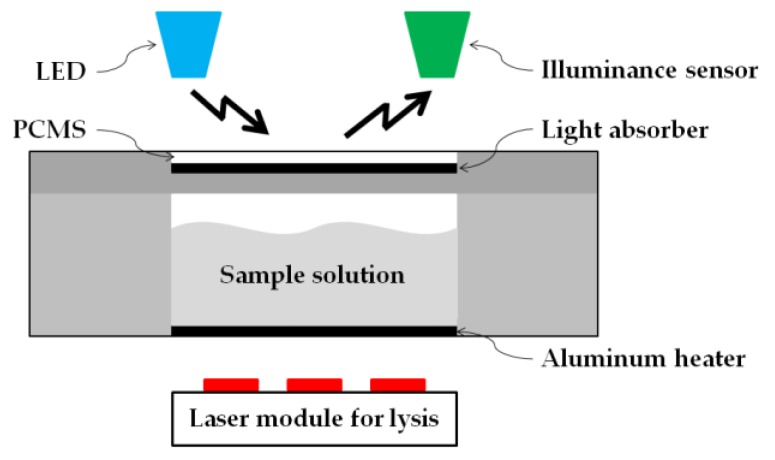
Schematic diagram that consists of phase change material sheet (PCMS) and illuminance sensor to measure the temperature of the lysis chamber being heated by laser module.

Automation of the heating process for cell lysis was one of the objectives of the proposed study. Since the lysis process is performed at 60 °C, the Accuprep^®^ Genomic DNA extraction kit manual (K-3032, Bioneer, Daejeon, Korea), PCM-60 (PCM60, CELSIUS, Seoul, Korea) was used in this study. Figure 2 illustrates the location of PCMS in the entire disc. To illustrate its function, a side-view schematic of PCMS is depicted in Figure 4.

Phase change material (PCM) is a substance with a high heat of fusion which, melting and solidifying at a certain temperature, is capable of storing and releasing large amounts of energy [33]. When this PCM is transformed into a sheet form, which name “PCMS”, with a thickness of few micrometers, its status becomes very sensitive to temperature change. PCM-60 implies that its phase change occurs at a temperature of 60 °C. PCM-60 appears opaque white at room temperature, but at temperatures above 60 °C, its phase changes to that of a transparent liquid. As shown in Figure 1, the laser module for lysis heating is arranged at the bottom of the disc. Increase in temperature of the heater, made of aluminum sheet, causes the temperature in the lysis chamber to rise. PCMS is placed at a distance of 0.2 mm from the lysis chamber to facilitate maximum heat transfer from the lysis chamber to the PCMS. A light absorber is located under the PCMS. Above the PCMS, there exists an LED that is always in the ON state and an illuminance sensor (Figure 1). In the solid, opaque phase, PCMS reflects all the light of the LED and directs it to the illuminance sensor. In the liquid, transparent of PCMS, all the incident LED light is directed towards the light absorber. Since there was no light entering the illuminance sensor, the value indicated by it becomes zero.

## 3. Results and Discussion

### 3.1. Disc Fabrication and Structure 

**Figure 5:**
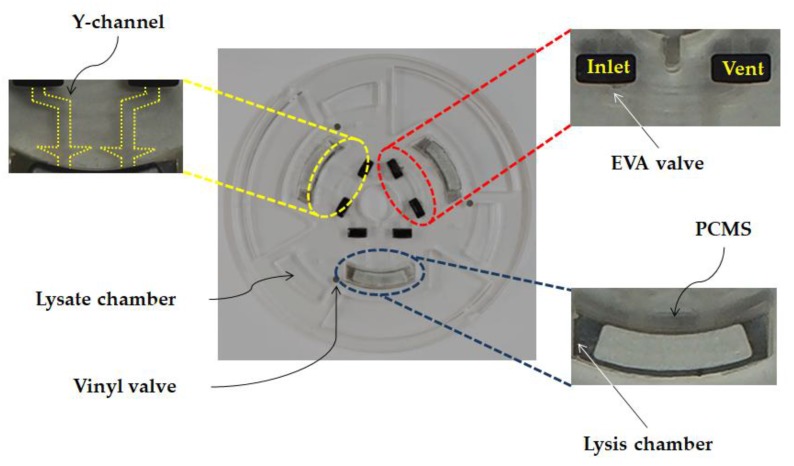
LOD design showing the detail of microfluidic layout and functions.

Polycarbonate was used as the main material in the fabrication of the disc in order to withstand the heat generated during the lysis heating process. Inlet and vent holes were drilled at innermost locations on the disc. The inlet and vent holes form essential parts for sample liquid injection. The EVA valve, Y-channel, lysis chamber, vinyl valve, and lysate chamber were arranged on the disc as depicted in Figure 5. The EVA valve was fabricated to prevent leakage of the injected liquid, while the Y-channel was designed to minimize leakage through the vent hole when the liquid is being injected into inlet hole. The lysis heating process was carried out in the lysis chamber thereby activating the reaction with PCMS constituting the top layer. After completion of the heating process, the vinyl valve was opened and the liquid was transferred to the lysate chamber by rotating the disc. Since a vent hole is also provided, the liquid in the lysate chamber can be taken out through use of a syringe.

### 3.2. Durability of Ethylene Vinyl Acetate Used as a Valve

**Figure 6:**
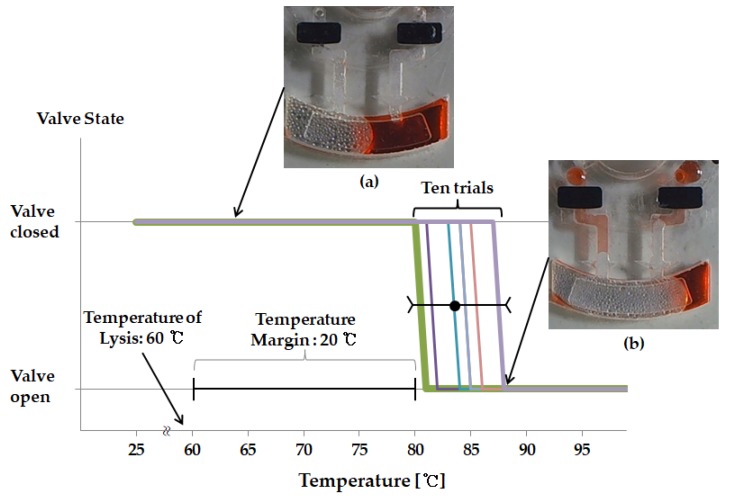
Graph showing how resistant the EVA valve is to pressure. The EVA valve has a temperature margin of 20 °C because the lysis operates at 60 °C. (**a**) When the EVA valve is closed, (**b**) when the EVA valve is softened and leaked by heat-induced pressure.

The lysis chamber was heated up to 60 °C by the laser module. When the liquid inside the chamber was heated up, the internal pressure increased, which caused a leak. Liquid leakage can occur at the inlet hole and vent hole, and it must be blocked. Therefore, the EVA valve was designed for each of the inlet holes and vent holes. The EVA valve, as shown in Figure 3, was able to withstand the pressure caused by thermal expansion. However, since the EVA valve also had a melting point, there was a limit to be able to withstand the pressure. Therefore, the experiment was conducted to measure the temperature limit for the EVA valve. To test the durability of the EVA valve, a red ink was injected through the inlet channel and the EVA valve was closed. After setting the initial temperature inside the lysis chamber to 25 °C, the temperature was increased until a leak of liquid occurred at the inlet holes or vent holes. The temperature was raised by 1 °C and then maintained for 5 min. The temperature was increased until the EVA valve could withstand the heat, and the limit temperature was checked. Durability test was repeated 10 times to demonstrate the reproducibility (Figure 6). As a result, the EVA valve was able to resist up to 84.1 °C (s.d. 2.7), which was sufficient to withstand the lysis heating temperature of 60 °C.

### 3.3. PCMS Performance

The process of increasing the temperature of the sample liquid to 60 °C depends on the situation—the uniformity of liquid distribution, ambient humidity, and temperature—in the lysis chamber. As such, it was impossible to automate the system by merely specifying the heating time. In order to achieve temperature changes in real time, the authors designed a system that could directly detect the temperature, and PCMS was used as the material of choice for this system on account of its temperature-dependent properties. In the proposed study, a temperature of 60 °C—since the target temperature of lysis is 60 °C—was maintained using PCM-60, which undergoes a phase change at 60 °C.

As the temperature in the lysis chamber increased, the heat generated was transferred to the PCMS causing it to gradually melt. At 60 °C, the PCMS was found to have completely liquefied with its phase changing to that of a transparent liquid. The black floor of the plate beneath the PCMS layer, which also served as a light absorber, was, thus, exposed. As a result, the amount of reflected light entering the illuminance sensor had gradually diminished. 

**Figure 7:**
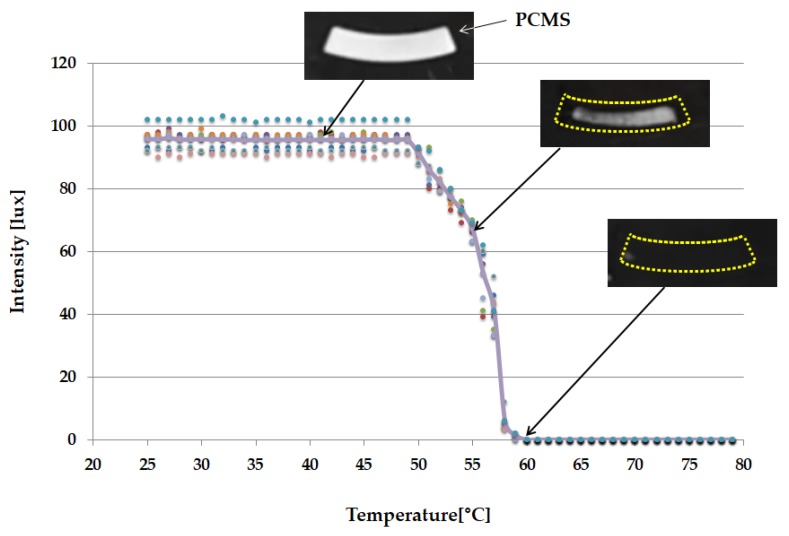
Graph showing the status of PCMS as the heating temperature increases. The PCMS is fully melt and transparent at around 60 °C.

Figure 7 depicts a graphical representation of this process. At 60 °C, the intensity value indicated by the illuminance sensor drops to zero. Consequently, it may be inferred that accurate lysis-chamber temperature sensing can be realized through combined operation of the PCMS, LED, and illuminance sensor. The temperature can, thus, be automatically controlled during the lysis process. The actual performance of cell lysis is not much sensitive to the temperature because the cell lysis operates well in the temperature range of 50 to 65 °C [34].

### 3.4. DNA Purification and Yield

**Figure 8:**
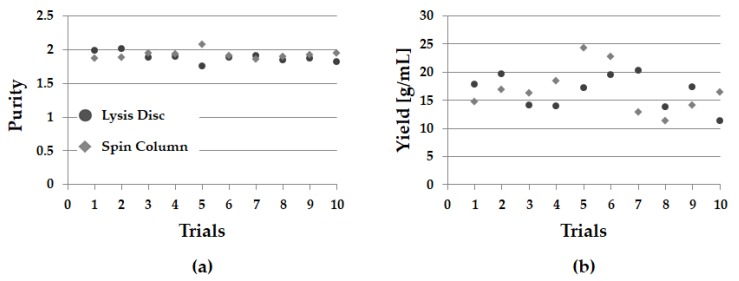
Graph showing the comparison of proposed LOD results and Spin Column results (**a**) DNA purity results. (**b**) DNA yield results.

To demonstrate performance of the proposed lysis disc, the lysis experiment inside the disc and the conventional method—spin column—were carried out concurrently. To see reproducibility, 10 replicate experiments were performed (Table 2). The average purity of DNA in the lysis disc and the spin column were 1.883 and 1.921, respectively, and the mean yield of DNA was 16.458 g/mL and 16.795 g/mL, respectively [27]. Despite the automation on the disc, we were able to achieve similar results as the conventional commercially uncomfortable method (Figure 8).

### 3.5. PCR Electrophoresis Results

**Figure 9:**
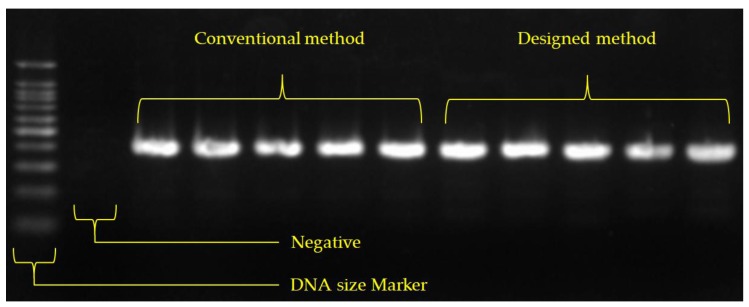
Photograph showing the electrophoresis comparison of the proposed LOD and existing spin column.

PCR was used as the amplification method in this study. In order to perform PCR, 10 μL of NobleZyme™ PCR Plus Premix 2× (E-220, Lot. No. E220S1402, Noble Bioscience, Hwaseong-si, Korea) was used and mixed with an upstream primer, 10 μM (2 μL); downstream primer, 10 μM (2 μL); DNA template (3 μL); and nuclease-free water (3 μL) as reaction components. The PCR protocol was based on the thermal cycling profile—denaturation at 95 °C for 2 min, 30 cycles at 95 °C for 45 s, at 52 °C for 45 s, at 72 °C for 60 s, and a final extension at 72 °C for 5 min. Electrophoresis procedures—using 2% agarose gel (Agarose, Cta. No. 32032, iNtRON Biotechnology, Seongnam-si, Korea ) with 0.5X TBE buffer (BT003, Bio solution, Seoul, Korea) at 100 V for 30 min—were used to obtain results of the PCR process.—were used to obtain results of the PCR process. The DNA size Marker (SiZer™-100, Intron biotechnology, iNtRON Biotechnology, Seongnam-si, Korea) was loaded with 5 μL on the first lane, and resultant solutions obtained from the conventional and proposed methods were loaded with 5 μL on 5 lanes each. 3X green gel stain (10 mL) was then placed on agarose in a dark room for 30 min, and stained DNA were finally observed using a fluorescence treatment (Figure 9).

## 4. Conclusions

A new heating system that serves to dissolve bacterial cells in DNA on an LOD platform is proposed in this paper. In order to prevent liquid leakage under increased pressure, an EVA valve with sufficient thermal durability has been designed. A dedicated laser module is used to heat the lysis chamber and a PCM array is used to maintain optimum chamber temperature. Additionally, a dedicated LED and illuminance sensor have been added to automate the entire heating process. 

Effectiveness of the proposed design has been demonstrated by measuring the purity and yield of the extracted DNA templates and their subsequent amplification using PCR. The results demonstrate that the lysis process was successfully performed and was fully automated. The authors expect that use of the proposed device would lead to user-friendly DNA analysis procedures in the near future. On the top of that, these experimental results tell us how stable the closed-loop system under PCM based temperature measurement is.

## Figures and Tables

**Table 1:** Summary of the experimental procedure of the LOD platform for DNA lysis.

Step	Procedure	Laser ON/OFF (s)	Time (s)
1	Load the sample into the main chamber	-	-
2	Spin the LOD-1500 rpm (To move the liquid remaining in the channel to the main chamber)	-	30
3	Ethylene Vinyl Acetate Valve closing	ON	60
4	Automated lysis system-reach to 60 °C	ON	90~100
5	Automated lysis system-retain 60 °C	2 s/4.5 s	600
6	Laser burst valve opening	ON	10
7	Spin the LOD-5000 rpm (the lysis solution moves to out chamber)	-	30

**Table 2:** Absorbance comparison of the proposed LOD and existing spin column.

Sample Number	Proposed LOD					Spin Column				
	A260	A280	A320	Purity	Yield	A260	A280	A320	Purity	Yield
1	0.385	0.209	0.029	1.982	17.800	0.301	0.164	0.006	1.864	14.755
2	0.419	0.222	0.027	2.011	19.600	0.359	0.201	0.022	1.881	16.830
3	0.295	0.163	0.014	1.888	14.045	0.350	0.192	0.025	1.946	16.270
4	0.285	0.154	0.008	1.895	13.870	0.374	0.196	0.007	1.938	18.375
5	0.351	0.205	0.008	1.747	17.130	0.495	0.244	0.010	2.072	24.265
6	0.401	0.219	0.010	1.876	19.555	0.471	0.255	0.016	1.903	22.710
7	0.411	0.218	0.006	1.912	20.260	0.284	0.166	0.028	1.850	12.805
8	0.291	0.165	0.016	1.839	13.730	0.242	0.134	0.014	1.894	11.375
9	0.377	0.216	0.030	1.865	17.340	0.306	0.171	0.024	1.920	14.105
10	0.249	0.148	0.024	1.815	11.245	0.347	0.187	0.017	1.944	16.455

(DNA purity = (A260 nm – A320 nm)/(A280 nm – A320 nm), DNA yield [g/mL] = (A260 nm – A320 nm) × 50 g/mL).

## References

[1] Stone H.A., Stroock A.D., Ajdari A. (2004). Engineering Flows in Small Devices: Microfluidics toward A Lab-on-a-Chip. Annu. Rev. Fluid Mech..

[2] Boyd-Moss M., Baratchi S., DiVenere M., Khoshmanesh K. (2016). Self-contained microfluidic systems: A review. Lab Chip.

[3] Nan L., Jiang Z., Wei X. (2014). Emerging microfluidic devices for cell lysis: A review. Lab Chip.

[4] Wang C.-H., Lai H.-C., Liou T.-M., Hsu K.-F., Chou C.-Y., Lee G.-B. (2013). A DNA methylation assay for detection of ovarian cancer cells using a HpaII/MspI digestion-based PCR assay in an integrated microfluidic system. Microfluid. Nanofluid..

[5] Chen L., Patrone N., Liang J.F. (2012). Peptide Self-Assembly on Cell Membranes to Induce Cell Lysis. Biomacromolecules.

[6] Marshall L.A., Wu L.L., Babikian S., Bachman M., Santiago J.G. (2012). Integrated Printed Circuit Board Device for Cell Lysis and Nucleic Acid Extraction. Anal. Chem..

[7] Zare R.N., Kim S. (2010). Microfluidic Platforms for Single-Cell Analysis. Annu. Rev. Biomed. Eng..

[8] Irimia D., Tompkins R.G., Toner M. (2004). Single-Cell Chemical Lysis in Picoliter-Scale Closed Volumes Using a Microfabricated Device. Anal. Chem..

[9] Sasuga Y., Iwasawa T., Terada K., Oe Y., Sorimachi H., Ohara O., Harada Y. (2008). Single-Cell Chemical Lysis Method for Analyses of Intracellular Molecules Using an Array of Picoliter-Scale Microwells. Anal. Chem..

[10] Jen C.-P., Hsiao J.-H., Maslov N.A. (2012). Single-Cell Chemical Lysis on Microfluidic Chips with Arrays of Microwells. Sensors.

[11] Stumpf F., Schwemmer F., Hutzenlaub T., Baumann D., Strohmeier O., Dingemanns G., Simons G., Sager C., Plobner L., von Stetten F. (2016). LabDisk with complete reagent prestorage for sample-to-answer nucleic acid based detection of respiratory pathogens verified with influenza A H3N2 virus. Lab Chip.

[12] Smith S., Sewart R., Becker H., Roux P., Land K. (2016). Blister pouches for effective reagent storage on microfluidic chips for blood cell counting. Microfluid. Nanofluid..

[13] Clingenpeel S., Clum A., Schwientek P., Rinke C., Woyke T. (2015). Reconstructing each cell’s genome within complex microbial communities—Dream or reality?. Front Microbiol..

[14] Dhawan M.D., Wise F., Baeumner A.J. (2002). Development of a laser-induced cell lysis system. Anal. Bioanal. Chem..

[15] Kong L.X., Parate K., Abi-Samra K., Madou M. (2015). Multifunctional wax valves for liquid handling and incubation on a microfluidic CD. Microfluid. Nanofluid..

[16] Al-Faqheri W., Thio T.H.G., Qasaimeh M.A., Dietzel A., Madou M., Al-Halhouli A. (2017). Particle/cell separation on microfluidic platforms based on centrifugation effect: A review. Microfluid. Nanofluid..

[17] La M., Park S.J., Kim H.W., Park J.J., Ahn K.T., Ryew S.M., Kim D.S. (2013). A centrifugal force-based serpentine micromixer (CSM) on a plastic lab-on-a-disk for biochemical assays. Microfluid. Nanofluid..

[18] Thiha A., Ibrahim F. (2015). A Colorimetric Enzyme-Linked Immunosorbent Assay (ELISA) Detection Platform for a Point-of-Care Dengue Detection System on a Lab-on-Compact-Disc. Sensors.

[19] Park J., Sunkara V., Kim T.H., Hwang H., Cho Y.K. (2012). Lab-on-a-Disc for Fully Integrated Multiplex Immunoassays. Anal. Chem..

[20] Uddin S., Ibrahim F., Sayad A., Thiha A., Pei K., Mohktar M., Thong K. (2015). A Portable Automatic Endpoint Detection System for Amplicons of Loop Mediated Isothermal Amplification on Microfluidic Compact Disk Platform. Sensors.

[21] Shen T., Huang L., Wang J. (2016). Analysis and experiment of transient filling flow into a rectangular microchannel on a rotating disk. Microfluid. Nanofluid..

[22] Soroori S., Rodriguez-Delgado J.M., Kido H., Dieck-Assad G., Madou M., Kulinsky L. (2016). The use of polybutene for controlling the flow of liquids in centrifugal microfluidic systems. Microfluid. Nanofluid..

[23] Godino N., Vereshchagina E., Gorkin R., Ducrée J. (2014). Centrifugal automation of a triglyceride bioassay on a low-cost hybrid paper-polymer device. Microfluid. Nanofluid..

[24] Lee B., Lee J.N., Park J.M., Lee J.G., Kim S., Cho Y.K., Ko C. (2009). A fully automated immunoassay from whole blood on a disc. Lab Chip.

[25] Chen J.M., Huang P.C., Lin M.G. (2008). Analysis and experiment of capillary valves for microfluidics on a rotating disk. Microfluid. Nanofluid..

[26] Lim D., Yoo J.C. (2017). Chemical Cell Lysis System Applicable to Lab-on-a-Disc. Appl. Biochem. Biotechnol..

[27] Choi M.S., Yoo J.C. (2015). Automated Centrifugal-Microfluidic Platform for DNA Purification Using Laser Burst Valve and Coriolis Effect. Appl. Biochem. Biotechnol..

[28] Chin C.D., Linder V., Sia S.K. (2007). Lab-on-a-chip devices for global health: Past studies and future opportunities. Lab Chip.

[29] Belgrader P., Young S., Yuan B., Primeau M., Christel L.A., Pourahmadi F., Northrup M.A. (2001). A Battery-Powered Notebook Thermal Cycler for Rapid Multiplex Real-Time PCR Analysis. Anal. Chem..

[30] Miao B., Peng N., Li L., Li Z., Hu F., Zhang Z., Wang C. (2015). Centrifugal Microfluidic System for Nucleic Acid Amplification and Detection. Sensors.

[31] Roy E., Stewart G., Mounier M., Malic L., Peytavi R., Clime L., Veres T. (2015). From cellular lysis to microarray detection, an integrated thermoplastic elastomer (TPE) point of care Lab on a Disc. Lab Chip.

[32] Lee B., Lee Y., Kim H.S., Kim T.H., Park J., Lee J.G., Cho Y.K. (2011). Fully integrated lab-on-a-disc for simultaneous analysis of biochemistry and immunoassay from whole blood. Lab Chip.

[33] Sharma A., Tyagi V.V., Chen C.R., Buddhi D. (2009). Review on thermal energy storage with phase change materials and applications. Renew. Sustain. Energy Rev..

[34] Nakajima H., Itoh K., Arakawa E., Inoue M. (1994). Degradation of a Polymerase Chain Reaction (PCR) Product by Heat-Stable Deoxyribonuclease (DNase) Produced from Yersinia enterocolitica. Microbiol. Immunol..

